# Effect of Lenalidomide Maintenance in Chronic Lymphocytic Leukemia: A Meta-Analysis and Trial-Sequential Analysis

**DOI:** 10.3390/curroncol29060339

**Published:** 2022-06-14

**Authors:** Tsung-Ying Yu, Hong-Jie Jhou, Po-Huang Chen, Cho-Hao Lee

**Affiliations:** 1 Division of Hematology and Oncology Medicine, Department of Internal Medicine, Tri-Service General Hospital, National Defense Medical Center, Taipei 11490, Taiwan; fishmen789@hotmail.com; 2 Department of Neurology, Changhua Christian Hospital, Changhua 500, Taiwan; 182902@cch.org.tw; 3 Department of Internal Medicine, Tri-Service General Hospital, National Defense Medical Center, Taipei 11490, Taiwan

**Keywords:** chronic lymphocytic leukemia, lenalidomide, maintenance therapy, meta-analysis

## Abstract

Chronic lymphocytic leukemia (CLL) is the most common lymphoproliferative disease in adults. Despite durable responses and sustained remission rates to frontline therapy, CLL is still incurable within standard therapy and eventually relapses. Maintenance therapies aim to achieve deep remission. However, the efficacy and safety of lenalidomide maintenance are still debated. Randomized controlled trials published before March 2022 were retrieved from databases. Primary outcomes were progression-free survival (PFS) and overall survival (OS). Trial sequential analysis examined analytical power in primary outcomes. Secondary outcomes were Grade 3–4 neutropenia, treatment discontinuation (TD), serious adverse events (SAE), and fatal adverse events (FAE). Hazard (HR) and odds ratios (ORs) with 95% confidence intervals (CIs) were calculated. Four articles (733 patients) met the selection criteria. Lenalidomide maintenance was associated with a statistically significant effect in prolonging PFS (HR, 0.43; 95% CI, 0.28–0.68; I^2^ = 57%) and higher proportion of SAE (OR 4.64; 95% CI 2.96–7.26; I^2^ = 0%) and exhibited no difference in OS (HR, 0.62; 95% CI, 0.29–1.30; I^2^ = 52%) observation/placebo. It showed no significant difference compared with observation/placebo regarding Grade 3–4 neutropenia (OR 2.30; 95% CI 0.84–6.28; I^2^ = 81%), TD (OR 0.76; 95% CI 0.29–1.99; I^2^ = 84%), and FAE (OR 0.86; 95% CI 0.28–2.63; I^2^ = 0%). Lenalidomide maintenance can prolong PFS in CLL. Further studies should verify its effect on OS.

## 1. Introduction

In the World Health Organization classification, chronic lymphocytic leukemia (CLL) is combined with small lymphocytic lymphoma as a mature B-cell lymphocytic neoplasm because the two seem to be the same disease with different clinical presentations [1]. CLL is the most common lymphoproliferative disease in adults in Western countries; it accounts for more than 20,000 new cases each year and approximately one-third of all leukemia cases [2]. The annual incidence of CLL in Western countries is 4.2 per 100,000 and gradually increases with age; about 90% of patients with CLL are older than 55 years, with a median age at diagnosis of 72 years [3]. CLL has a diverse disease process and prognosis. The International Prognostic Index for CLL classifies 5-year overall survival (OS) into four groups, ranging from 93.2% to 23.3% for low risk to very high risk [4]. The median OS ranges from 18 months to more than 10 years depending on two clinical staging criteria: the Rai system (used in North America) [5] and the Binet system (used in Europe) [6]. These criteria classify patients into three similar groups: low risk (Rai K; Binet A), intermediate risk (Rai I, II; Binet B), and high risk (Rai III/IV; Binet C) of disease [7].

Several predictive markers are associated with prognosis. Immunoglobulin heavy chain variable region (IGHV) gene mutation is associated with high response rates and improved overall survival (OS) in patients receiving fludarabine, cyclophosphamide, and rituximab (FCR) [8,9,10]. CLL could be classified into four prognostic subgroups: high-risk (TP53 and/or BIRC3 abnormalities), intermediate-risk (NOTCH and/or SF3B1 mutations and/or del(11q)), low-risk (trisomy 12 and wild-type for all genetic lesions), and very low risk (del(13q) only). The 10-year survival rates for the four subgroups were 29%, 37%, 57%, and 69%, respectively [11,12]. Among the cell surface markers detected by flow cytometry or immunohistochemistry, CD38 expression and/or ZAP-70 were associated with shorter PFS and OS outcomes [13,14,15,16]. Early-stage patients are not treated with chemotherapy until they present symptoms or rapid disease progression [17]. The standard therapy for CLL includes monochemotherapy (i.e., fludarabine, chlorambucil, bendamustine) or polychemotherapy (i.e., cyclophosphamide, doxorubicin, vincristine, and prednisolone; fludarabine combined with cyclophosphamide), which is usually combined with anti-CD20 monoclonal antibodies (i.e., rituximab, ofatumumab) [18,19]. Novel targeted therapies (e.g., Bruton’s tyrosine kinase (BTK) inhibitor, ibrutinib; phosphoinositide 3-kinase (PI3K) inhibitor, idelalisib; and B-cell lymphoma-2 protein (BCL-2) inhibitor, venetoclax) are rapidly changing the treatment circumstance of CLL [20,21]. Despite durable responses and sustained remission rates to first-line therapy, patients often relapse within 5 years after the initial treatment [22,23]. Some patients even have markedly reduced OS due to early progression and poor response to salvage therapy [24]. Chemoimmunotherapy is not recommended because del(17p)/TP53 mutations are associated with low response rates. The PFS benefit of acalabrutinib +-obinutuzumab was observed in CLL patients with del(17p) or TP53 mutations, IGHV-unmutated, and IGHV-mutated CLL [25]. In patients with del(17p) or TP53 mutations, the undetectable MRD rate and PFS rate were significantly higher with venetoclax + obinutuzumab than those with chlorambucil + obinutuzumab [26].

Although kinase inhibitors can improve the outcomes, not all patients have access to these novel drugs. Second-line treatment with kinase inhibitors, including BTK, PI3K, and BCL-2 inhibitors, guide to partial and prolonged response in most patients with refractory or relapsed disease, but complete responses with no minimal residual disease (MRD) are rare [9,27,28].

The concept of maintenance therapy is not new in hematological malignancy. By delaying disease progression and improving survival, it was a successful approach in all incurable mature B-cell tumors, including follicular lymphoma, mantle cell lymphoma, and multiple myeloma [29]. Since 2011, a review on the maintenance therapy for B-CLL indicated that the maintenance therapy of rituximab and lenalidomide had a potential role in deep treatment response [30]. Maintenance therapies aim to reach deep remission and extend the period of disease quiescence. Previous network meta-analysis had shown that maintenance therapies achieved superior effect in prolonging progression-free survival (PFS) compared without intervention. Moreover, the therapy did not significantly increase the risk of overall serious adverse events (SAE) [31].

Lenalidomide, an immunomodulatory agent, has been used to treat multiple myeloma, certain subtypes of non-Hodgkin lymphoma such as mantle cell lymphoma, and CLL, the last as part of clinical trials [32,33,34,35]. On the basis of a review of the trial data, it appears that lenalidomide therapy causes no specific immune dysfunction that would increase the risk of opportunistic infections in these patients [36]. Therefore, routine antimicrobial prophylaxis is not usually offered to patients receiving this agent. However, this agent is not approved for the treatment of CLL patients outside clinical trials [37].

Lenalidomide is a 4-amino-glutamyl analogue of thalidomide that removes neurologic side effects and neuropathy, and has activity against various hematological and solid malignancies. It is FDA-approved for the clinical treatment of multiple myeloma and chromosome 5q deletion myelodysplastic syndrome. It has been shown to be an immunomodulator affecting the immune system, and to have antiangiogenic properties [38]. Lenalidomide has a special mechanism of action: it can target cancer cells and modulate or interrupt several interactions of CLL cells and elements in their microenvironment [39,40,41], which can stimulate leukemia or have an impact on survival [42,43]. The efficacy and safety of lenalidomide as maintenance therapy for CLL are inconsistent within individual studies and still under debate. Moreover, it remains unknown whether previous meta-analyses established sufficient statistical power to draw a firm conclusion. Therefore, we performed a systematic review and meta-analysis of all available Phase II and III randomized controlled trials (RCTs) and applied trial sequential analysis (TSA) to examine the statistical power.

## 2. Methods

### 2.1. Search Strategy

We reported and prepared this study on the basis of the Preferred Reporting Items for Systematic Reviews and Meta-Analyses statement [44] for performing systematic reviews and meta-analyses of RCTs (Supplementary Information S1). The protocol used in this systematic review was registered in the Open Science Framework (https://osf.io/wpnh5/ (accessed on 23 April 2022)).

### 2.2. Study Selection

We identified potential studies with a systematic search of the PubMed, Embase, and Cochrane Library databases from inception until March 2022 without language restrictions. The following search terms were used for the search in databases: “chronic lymphocytic leukemia”, “maintenance”, “consolidation”, and “lenalidomide” (Supplementary Information S2), along with possible relevant keywords. The references in the relevant eligible studies were also manually searched.

### 2.3. Eligibility Criteria

All studies including patients with a definitive diagnosis of CLL received frontline treatment and achieved at least a partial response, and those that further accepted subsequent maintenance therapy using lenalidomide were included. The diagnosis of CLL was made according to the criteria of the International Workshop on Chronic Lymphocytic Leukemia and also made according to the World Health Organization classification of lymphoid neoplasms, 2016 revision [1,45]. RCTs that enrolled the comparisons of maintenance lenalidomide and placebo or observation were included in our studies.

Two reviewers (PHC and TYY) independently screened the studies using the selection criteria. Disagreements regarding selection process for each individual study were resolved by group consensus.

### 2.4. Outcome Measurement

Both PFS and OS were the primary outcomes. The secondary outcomes included Grade 3 and 4 neutropenia, treatment discontinuation (TD), SAE, and fatal adverse events (FAE). PFS was the length of time from study entry until objective disease progression or death. OS was the period from study enrolment until all causes of death. Grade 3 and 4 neutropenia was defined as the definition of Common Terminology Criteria for Adverse Events (CTCAE) [46]. TD was defined as TD from any cause at any time after participants had been randomized to intervention or comparator groups. SAE comprised Grade 3 and 4 adverse events according to CTCAE [47]. FAE was deaths related to adverse events at any time from the beginning of treatment to the end to 28 days after the study treatment had been stopped.

### 2.5. Data Extraction and Management

Each reviewer extracted the following data using a standardized data collection form: (1) study information, (2) characteristics of participants, and (3) treatment-related data. The corresponding authors were contacted when studies did not provide enough data to estimate the effect size. The methodological quality of the included studies was assessed according to the Cochrane Handbook for Systematic Reviews of Interventions. We produced the risk-of-bias graphs using Review Manager 5.3 software to critically appraise the studies (The Cochrane Collaboration, 2014; The Nordic Cochrane Centre, Copenhagen, Denmark) (Supplementary Information S3).

We measured the time-to-event outcomes (PFS and OS) using hazard ratios (HRs), standard errors, and number of randomized patients. For dichotomous outcomes (Grade 3 and 4 neutropenia, TD, SAE, and FAE), we extracted the number of randomized patients, number of analyzed patients, and number of events per arm. We only used intention-to-treat (ITT) design even if recommended larger effect sizes were possible per protocol design [48].

### 2.6. Statistical Analysis

We followed the proposal of the Cochrane Handbook for Systematic Reviews of Interventions to analyze the data [49]. The meta-analysis was conducted in a fixed-effect model or DerSimonian–Laird random-effects model using the inverse-variance method. The dichotomous outcomes were estimated with odds ratios (ORs) with 95% confidence intervals (CIs), and time-to-event outcomes were estimated with HRs with their 95% CIs. Heterogeneity was assessed with the Cochran Q test and I^2^ statistics [50]. A *p*-value < 0.1 or the I^2^ value > 50% indicated significance [51]. Using fixed or random effects was interpreted according to statistical heterogeneity. If there was between-study heterogeneity, and the I-squared was low, we would use the fixed-effect model. On the other hand, if heterogeneity was high, we would use the random-effects model.

Subgroup analysis was performed with the high level of minimal residual disease (MRD) of 0.01%, which is clinically important, higher than one CLL cell per 10,000 leukocytes, measured at least two months after the last treatment. The bias from small study effects was examined using funnel plots and the Egger test. *p*-values < 0.05 meant significance [52].

We used the “metafor” [53] and “meta” packages of R software version 4.0.1 for all analyses [54]. A *p*-value < 0.05 indicated statistical significance.

### 2.7. Trial Sequential Analysis (TSA)

Sparse data and repetitive testing in the meta-analysis may lead to a risk of Type 1 and 2 errors. Even if meta-analysis is statistically significant in the outcomes, it might be insufficient statistical power to estimate the true effects [55]. The advantages of TSA include recalculating the sample size required or stopping further trials when the intervention has no benefits [56]. Furthermore, it can present whether meta-analysis has enough statistical significance for adequate power. A TSA model was produced assuming a 5% (two-sided α = 0.05) Type 1 error and 80% statistical power on the basis of the O’Brien–Fleming alpha-spending function. We assumed a relative risk reduction of 30% for PFS and OS. We used the Stata 16.0 metacumbounds software package (StataCorp LLC, College Station, TX, USA) for fixed-effect TSA [57].

## 3. Results

### 3.1. Search Results

**Figure 1:**
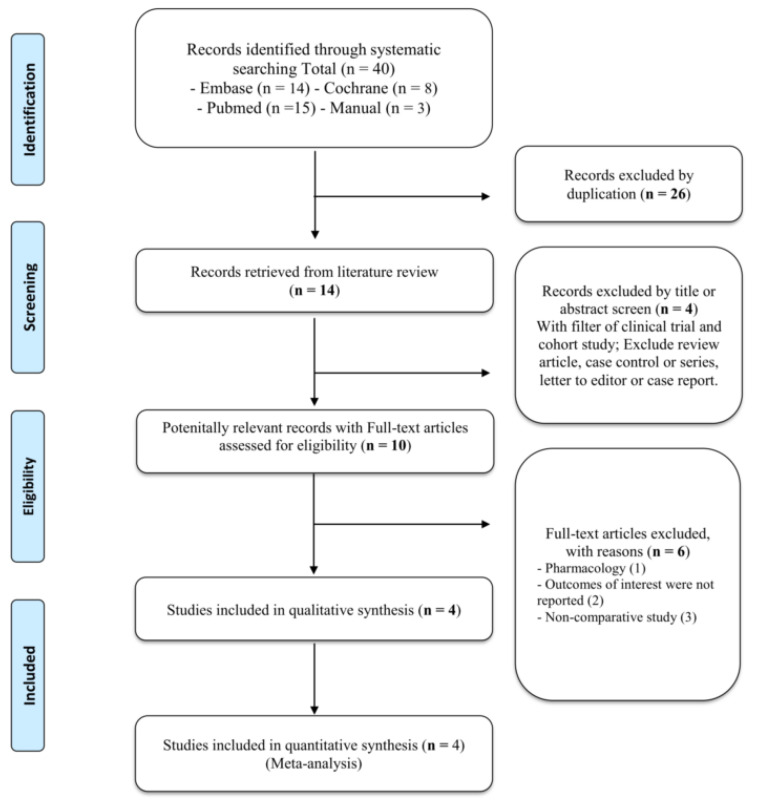
Flow diagram of the identification process for eligible studies.

The systematic search identified 40 articles (Figure 1). After the removal of 26 duplicate records, the titles and abstracts of fourteen were screened. Of the 10 potentially eligible studies, 6 articles were excluded after full-text review. Therefore, four studies met the inclusion criteria in the review.

### 3.2. Characteristics of Included Studies

The baseline characteristics of the studies included in meta-analysis are summarized in Table 1; all of which were Phase II or III RCTs using ITT design to estimate the effect size. The 4 RCTs comprised 733 patients (mean age: 59–64 years; median follow-up duration: 17.0 to 73.0 months). Three trials enrolled patients using only first-line therapy [58,59,60], and the other one involving patients receiving at least two lines of therapy [61]. The complete response rate was between 23.9% and 56.0%, whereas the partial response rate ranged from 37.0% to 76.1%.

### 3.3. Primary Outcomes–PFS

**Figure 2:**
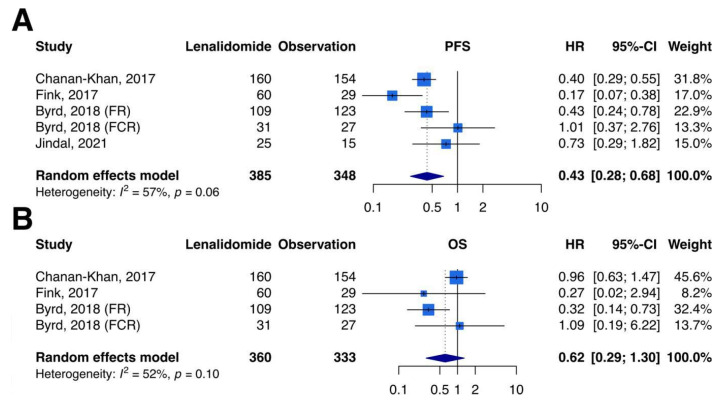
Meta-analysis of primary outcomes regarding (**A**) progression-free survival and (**B**) overall survival. PFS, progression-free survival; OS, overall survival; HR, hazard ratio; CI, confidence interval. (**A**) Meta-analysis of progression-free survival. Analysis included data from 5 comparisons with a total of 385 patients receiving lenalidomide maintenance therapy and 348 patients receiving observation alone. The *p*-value for heterogeneity was 0.06, and I-squared for heterogeneity was 57%, indicating moderate heterogeneity. We adopted a random-effects model, and lenalidomide maintenance therapy was associated with a statistically significant effect in the prolongation of PFS (HR, 0.43; 95% CI, 0.28–0.68). (**B**) Meta-analysis of overall survival. Analysis included data from 4 comparisons with a total of 360 patients receiving lenalidomide maintenance therapy and 333 patients receiving observation alone. The *p*-value for heterogeneity was 0.10, and I-squared for heterogeneity was 52%, indicating moderate heterogeneity. We adopted a random-effects model, and lenalidomide maintenance showed no difference in OS compared with the observation (HR, 0.62; 95% CI, 0.29–1.30).

**Figure 3:**
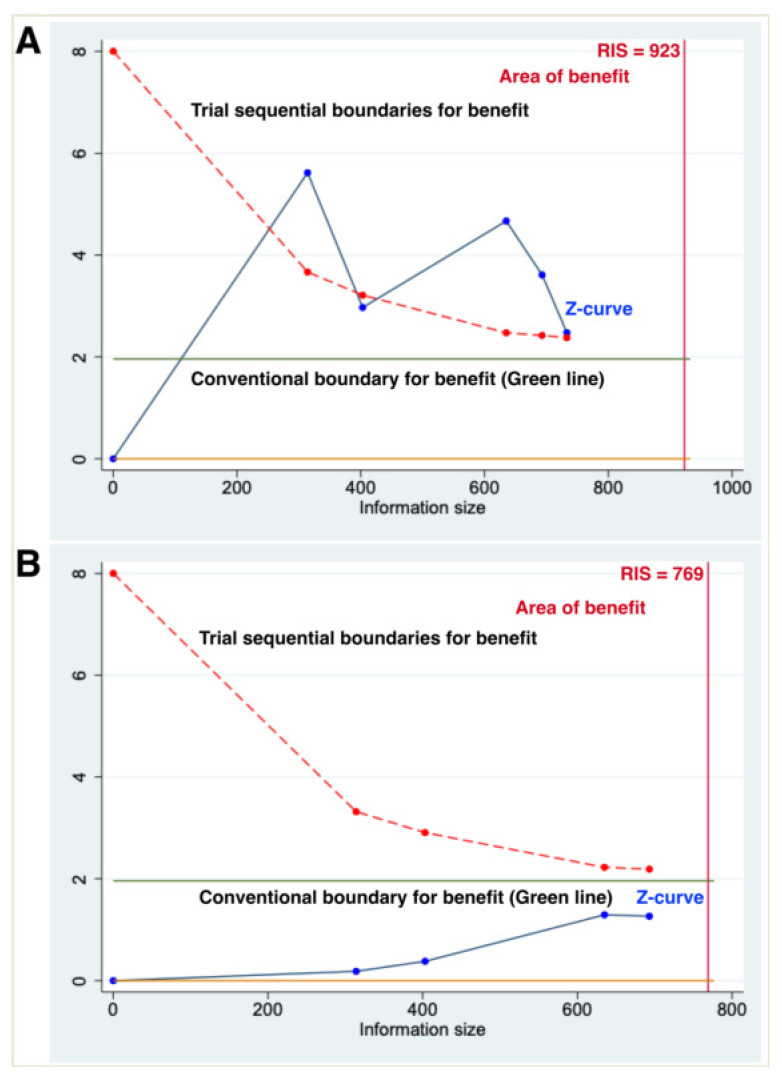
Trial sequential analysis (TSA) of primary outcomes regarding (**A**) progression-free survival and (**B**) overall survival. X axis indicates the information size referring to cumulative patient numbers; Y axis indicates the Z-score; green horizontal lines indicate conventional boundaries; red sloping lines at the top left-hand corners indicate the trial sequential boundaries as the TSA threshold for statistical significance. Red vertical full line indicates the required information size (RIS). Blue solid line indicates the cumulative Z-curve. (**A**) Trial sequential analysis of progression-free survival. The cumulative number of patients did not exceed the number of 923 patients, but the Z-curves surpassed the significance boundary in favor of lenalidomide maintenance therapy, suggesting a conclusive result. (**B**) Trial sequential analysis of overall survival. The cumulative patient numbers did not surpass the required patient number of 769, and the Z-curves did not surpass any significance boundary, indicating an inconclusive result of meta-analysis.

PFS analysis included 4 studies and 5 comparisons with 733 patients. Compared with the observation, lenalidomide maintenance therapy was associated with a statistically significant effect in the prolongation of PFS (random-effects HR, 0.43; 95% CI, 0.28–0.68; Cochran Q *p*-value: 0.06, I^2^ = 57%, Figure 2A). In the TSA, the cumulative patient numbers did not exceed the required information size of 923, but the Z-curves exceeded the significance boundary in favor of lenalidomide maintenance therapy, suggesting a conclusive result of meta-analysis and indicating convincing statistical evidence (Figure 3A).

### 3.4. Primary Outcomes–OS

OS analysis included 3 studies and 4 comparisons with 693 patients. Compared with the observation, lenalidomide maintenance showed no difference in OS (random-effects HR, 0.62; 95% CI, 0.29–1.30; Cochran Q *p*-value: 0.10, I^2^ = 52%, Figure 2B). TSA showed an inconclusive result because the cumulative number of patients did not surpass the required information size of 769, and the Z-curves did not surpass any significance boundary, indicating an inconclusive result of meta-analysis. Further studies are necessary for providing convincing statistical evidence (Figure 3B).

### 3.5. Secondary Outcomes

The analysis of Grade 3–4 neutropenia included 4 studies and 5 comparisons with 733 patients. Compared with the observation, lenalidomide maintenance showed no difference in Grade 3–4 neutropenia (random-effects OR 2.30; 95% CI 0.84–6.28; Cochran Q *p*-value < 0.01, I^2^ = 81%, Table 2, Supplementary Information S4).

The analysis of TD included 4 studies and 5 comparisons with 733 patients. Compared with the observation, lenalidomide maintenance showed no difference in TD (random-effects OR 0.76; 95% CI 0.29–1.99; Cochran Q *p*-value < 0.01, I^2^ = 84%, Table 2, Supplementary Information S4).

The analysis of SAE included 2 studies and 2 comparisons with 400 patients. Compared with the observation, lenalidomide maintenance was associated with a higher proportion of SAE (fixed-effect OR 4.64; 95% CI 2.96–7.26; Cochran Q *p*-value: 0.34, I^2^ = 0%, Table 2, Supplementary Information S4).

The analysis of FAE included 4 studies and 5 comparisons with 733 patients. Compared with the observation, lenalidomide maintenance showed no difference in FAE (fixed-effect OR 0.86; 95% CI 0.28–2.63; Cochran Q *p*-value: 0.66, I^2^ = 0%, Table 2, Supplementary Information S4).

### 3.6. Subgroup Analysis of PFS in the High-MRD Group

The analysis of PFS in the high-MRD group included 2 studies with 47 patients. Compared with the observation, lenalidomide maintenance therapy was associated with a more statistically significant effect in prolonging PFS (fixed-effect HR, 0.18; 95% CI, 0.07–0.46; Cochran Q *p*-value: 0.80, I^2^ = 0%) (Supplementary Information S5).

### 3.7. Publication Bias

There was no publication bias in the meta-analysis (Supplementary Information S6). However, this should be explained with caution because there were fewer than 10 studies used in this meta-analysis to assess publication bias [62].

## 4. Discussion

To the best of our knowledge, this is the first meta-analysis with TSA to evaluate the relative efficacy and safety of lenalidomide maintenance therapy for CLL patients. The results demonstrate that this therapy achieved a superior effect in prolonging PFS compared without intervention. OS did not significantly differ between the two groups; however, the TSA indicated that the statistical power was inconclusive. On the other hand, the administration of lenalidomide maintenance therapy was associated with a higher proportion of SAE compared with that without intervention. However, there was no significant difference between lenalidomide maintenance therapy and observation regarding Grade 3–4 neutropenia, TD, and FAE.

Several large RCTs [58,59,61] included in our study reported the significant prolongation of PFS in patients with CLL who received lenalidomide maintenance. In the CLLM1 study (NCT01556776), 89 patients with CLL were randomly assigned (2:1) to receive 5 mg of lenalidomide for maintenance or a placebo. The HR for PFS was 0.168 (95% CI, 0.074–0.379) with a median follow-up of 17.9 months. The median PFS was not reached (95% CI, 32.3 to not evaluable) in the lenalidomide group and 13.3 months (95% CI, 9.9–19.7) in the placebo group. However, due to insufficient candidates, study recruitment was stopped early [58]. The Phase III CONTINUUM trial (NCT00774345) found that lenalidomide maintenance therapy prolonged PFS without influencing potential subsequent lines of therapy. In this study, patients had reached complete or partial responses to second-line therapy. Then, they were randomly assigned to maintenance therapy with lenalidomide (160 patients) starting at 2.5 mg per day and titrating up to 5 or 10 mg as tolerated or to a placebo group (154 patients). Lenalidomide reduced more than 50% of the risk of progression compared with placebo (HR, 0.40; 95% CI, 0.29–0.55), but there was no difference in OS during a follow-up [61]. The GALGB 10,404 study revealed significantly longer median PFS in non-del(11q) patients who had received fludarabine and rituximab (FR) plus lenalidomide maintenance (60.7 months; 95% CI, 44.8–71.3) compared with FR alone (43.5 months; 95% CI, 32.8–50.2). For del(11q) patients, the median PFS with fludarabine, cyclophosphamide, and rituximab (FCR) plus lenalidomide maintenance (41.2 months; 95% CI, 25.7–50.7) was longer than that with FCR alone (35.5 months; 95% CI, 21.8–65.5) [59].

Another randomized control study released in the ASH abstract report, the CLL6 Residuum Study of the Australasian Leukemia and Lymphoma Group and the French Innovative Leukemia Organization, was not enrolled in our meta-analysis because it focused on flow analysis for MRD. CLL6 analyzed the role of lenalidomide as consolidation therapy in patients following first-line treatment for CLL with residual MRD. In this analysis, there was a clear trend for improved control of MRD in the lenalidomide group [63].

In our meta-analysis, the benefits of lenalidomide maintenance therapy in PFS were not transferred to OS. Furthermore, the results of TSA showed that the OS outcome still remained inconclusive due to the small sample size. The effects on survival are difficult to determine in patients with CLL due to the long follow-up needed in such an indolent disease and the puzzling effect of continued treatments [64]. The European Medicines Agency recommends using time to second objective disease progression to help in understanding the connection of meaningful improvements in PFS when OS cannot be measured [65]. The time to second objective disease progression is calculated in the intent-to-treat population as the time from randomization to the date of disease progression or death after second-line therapy [66]. It is useful to rule out any potentially adverse effect of first-line therapy on the efficacy of subsequent therapeutic regimens [67]. It can also be a surrogate endpoint providing insights into the effects of maintenance treatment on the efficacy of next-line therapy, and thus should be further explored.

In the subgroup of PFS in high-MRD patients, which was performed on bone marrow aspirate samples using eight-color flow cytometry with a detection threshold of >0.01, a significant trend toward improved PFS was observed with lenalidomide. There is strong evidence that MRD-negative status is the most important predictor of the final outcome in patients with previously untreated and relapsed CLL [68]. A previous study on CLL patients receiving rituximab maintenance therapy suggested that the analysis of bone marrow MRD after fludarabine-based induction may be a strong predictor of outcomes after maintenance therapy and a valuable tool to identify patients at high risk of relapse, influencing further treatment strategies [69]. Another study assessed PFS and OS in MRD-positive patients who had received anti-CD20 and concluded that maintenance therapy for MRD-positive patients increases PFS and OS to the level of MRD-negative patients, which is consistent with the results of our subgroup analysis. Maintenance therapy may be a means of controlling or eradicating MRD. The result provides an opinion into the use of induction FCR treatment followed by maintenance therapy as a treatment strategy in patients with CLL and detectable MRD. The treatment algorithm needs to be further investigated to confirm the initial results.

The study still had several limitations. Clinical heterogeneities existed from participant characteristics and previous interventions across trials, such as differences in the underlying disease severity (baseline Rai/Binet status, cytogenetic aberration, and IGHV status), previous treatment regimen, posttherapy response, timing to maintenance therapy, and regimens of maintenance therapy. Furthermore, despite several studies focusing on maintenance lenalidomide therapy, a well-defined optimal dose and biomarkers for identifying responders or toxic events beforehand are still lacking. Therefore, the results of this meta-analysis should be interpreted with caution.

## 5. Conclusions

Our meta-analysis of the current evidence suggests that the use of lenalidomide as maintenance therapy could prolong PFS and significantly reduce the risk of disease progression compared with no intervention. Moreover, TSA confirmed the statistical power of the meta-analysis. Compared with observation, lenalidomide maintenance showed no difference in OS. Further studies are warranted to justify the conclusion of OS. Regarding safety concerns, lenalidomide maintenance therapy was associated with a higher proportion of SAE, but there was no significant difference between lenalidomide maintenance therapy and observation regarding Grade 3–4 neutropenia, TD, and FAE. More high-quality randomized control trials should be conducted to provide reasonably conclusive evidence of the benefit to patients.

## Figures and Tables

**Table 1:** Basic characteristics of included randomized trials.

Trial Name/Registration Code/PublicationYear	Study Design	Treatment Comparison/Experimental Regimen		Cases	Mean Age	Follow Up (Range)/Analysis	Frontline Cases (%)/FCR Regimen(%)	CR/PR (%)	Frontline Regiments
CONTINUUM (NCT00774345) Chanan-Khan, 2017 [61]	Phase III, DB, MC, RCT	Lenalidomide vs. placebo	Oral 2.5 mg/daily (maximal 5 mg/daily)	160 vs. 154	63 vs. 63	31.5 months (18.9–50.8)/ITT	28%/98.9%	23.9%/76.1%	FCR, chlorambucil, alemtuzumab
CLLM1 (NCT01556776) Fink, 2017 [58]	Phase III, DB, MC, RCT	Lenalidomide vs. placebo	Oral 5 mg/daily (maximal 15 mg/daily)	60 vs. 29	64 vs. 64	17.9 months (9.1–28.1)/ITT	100%/22.1%	39.3%/60.7%	FCRB
CALGB 10404 (NCT00602459) Byrd, 2018 [59]	Phase II, OP, MC, RCT	Lenalidomide vs. observation (FR group)	Oral 5 mg/daily (maximal 10 mg/daily)	109 vs. 123	62 vs. 61	73.0 months (2.0–112.0)/ITT	100%/0%	32.0%/37.0%	FR
		Lenalidomide vs. observation (FCR group)		31 vs. 27	59 vs. 60	73.0 months (2.0–112.0)/ITT	100%/100%	35.0%/39.0%	FCR
Jindal et al. (CTRI/2018/07/014716) Jindal, 2021 [60]	Phase II, OP, SC, RCT	Lenalidomide vs. observation	Oral 5 mg/daily (maximal 10 mg/daily)	25 vs. 15	60 vs. 62	22.0 months (4.0–30.0)/ITT	100%/20%	56.0%/66.7%	BR, FCR, R-Chlorambucil, Chlorambucil

CR: complete response, PR: partial response, OP: open label, DB: double blind, MC: multiple centers, SC: single center, RCT, randomized controlled trial, ITT: intention to treat, FCR: fludarabine, cyclophosphamide, and rituximab, FCRB: fludarabine, cyclophosphamide, rituximab, and bendamustine, FR: fludarabine and rituximab, BR: bendamustine and rituximab, R-Chlorambucil: rituximab and chlorambucil.

**Table 2:** Secondary outcomes of meta-analysis.

Outcome	Comparison Trial Number (N)	Patients Number (N)	Measurement (95% CIs)	Cochran Q *p*-Value for Heterogeneity	I^2^ (%)
Grade 3–4 neutropenia	4 *	733	Random-effects, OR, 2.30 (0.84 to 6.28)	<0.01	81%
Treatment discontinuation	4 *	733	Random-effects, OR, 0.76 (0.29 to 1.99)	<0.01	84%
Serious adverse events	2	400	Fixed-effect, OR, 4.64 (2.96 to 7.26)	0.34	0%
Fatal adverse events	4 *	733	Fixed-effect, OR, 0.86 (0.28 to 2.63)	0.66	0%

OR, odds ratio; CIs, confident intervals; N, number, * two data from one trial (CALGB 10404).

## Data Availability

The data presented in this study are available in this article and supplementary material.

## Supplementary Materials

The following supporting information can be downloaded at: https://www.mdpi.com/article/10.3390/curroncol29060339/s1. Supplementary Information S1: PRISMA Checklist. Supplementary Information S2: Search strategies and detailed records. Supplementary Information S3: Assessment of risk of bias. Supplementary Information S4: Secondary outcomes of meta-analysis. Supplementary Information S5: Subgroup analysis of outcomes. Supplementary Information S6: Funnel plots and Egger’s test.

## Abbreviations

BCL-2	B-cell lymphoma-2 protein
BR	Bendamustine and Rituximab
BTK	Bruton’s tyrosine kinase
CIs	Confidence intervals
CLL	Chronic lymphocytic leukemia
CR	Complete response
DB	Double blind
FAE	Fatal adverse events
FCR	Fludarabine, cyclophosphamide, and rituximab
FCRB	Fludarabine, cyclophosphamide, rituximab, and bendamustine
FDA	Food and Drug Administration
FISH	Fluorescence in situ hybridization
FR	Fludarabine and rituximab
TD	treatment discontinuation
HR	Hazard ratio
IGHV	Immunoglobulin heavy chain variable region
ITT	Intention to treat
MC	Multiple centers
MRD	Minimal residual disease
OP	Open label
ORs	Odds ratios
OS	Overall survival
PFS	Progression-free survival
PI3K	Phosphoinositide 3-kinase
PR	Partial response
R-Chlorambucil	Rituximab and chlorambucil
RCTs	Randomized controlled trials
SAE	Serious adverse events
SC	Single center
TSA	Trial sequential analysis
