# Supplementary material for: Effect of Lenalidomide Maintenance in Chronic Lymphocytic Leukemia: A Meta-Analysis and Trial-Sequential Analysis
Source: Curr Oncol. 2022 Jun 14;29(6):4245–59. doi: 10.3390/curroncol29060339 (PMC9222174; doi:10.3390/curroncol29060339)
Supplement: Supplementary file 1 [file curroncol-29-00339-s001.zip › curroncol-1717606-supplementary.pdf]

# Supplementary Information

## A Meta-analysis and Trial sequential analysis

### Contents

Supplementary Information S1 PRISMA Checklist

Supplementary Information S2 Search strategy

Supplementary Information S3 Assessment of risk of bias

Supplementary Information S4 Secondary outcomes of meta-analysis.

Supplementary Information S5 Subgroup analysis of outcomes

Supplementary Information S6 Funnel plots and Egger's test

## Supplementary Information S1 - PRISMA Checklist

| Section/topic | Item No | Checklist item | Reported on page No |
|---|---|---|---|
| **Title** | | | |
| Title | 1 | Identify the report as a systematic review, meta-analysis, or both | 1 |
| **Abstract** | | | |
| Structured summary | 2 | Provide a structured summary including, as applicable, background, objectives, data sources, study eligibility criteria, participants, interventions, study appraisal and synthesis methods, results, limitations, conclusions and implications of key findings, systematic review registration number | 3 |
| **Introduction** | | | |
| Rationale | 3 | Describe the rationale for the review in the context of what is already known | 4 |
| Objectives | 4 | Provide an explicit statement of questions being addressed with reference to participants, interventions, comparisons, outcomes, and study design (PICOS) | 5 |
| **Methods** | | | |
| Protocol and registration | 5 | Indicate if a review protocol exists, if and where it can be accessed (such as web address), and, if available, provide registration information including registration number | 5 |
| Eligibility criteria | 6 | Specify study characteristics (such as PICOS, length of follow-up) and report characteristics (such as years considered, language, publication status) used as criteria for eligibility, giving rationale | 6 |
| Information sources | 7 | Describe all information sources (such as databases with dates of coverage, contact with study authors to identify additional studies) in the search and date last searched | 5-6 |
| Search | 8 | Present full electronic search strategy for at least one database, including any limits used, such that it could be repeated | 6 |
| Study selection | 9 | State the process for selecting studies (that is, screening, eligibility, included in systematic review, and, if applicable, included in the meta-analysis) | 6 |
| Data collection process | 10 | Describe method of data extraction from reports (such as piloted forms, independently, in duplicate) and any processes for obtaining and confirming data from investigators | 6 |
| Data items | 11 | List and define all variables for which data were sought (such as PICOS, funding sources) and any assumptions and simplifications made | 7 |

| Section/topic | Item No | Checklist item | Reported on page No |
|---|---|---|---|
| Risk of bias in individual studies | 12 | Describe methods used for assessing risk of bias of individual studies (including specification of whether this was done at the study or outcome level), and how this information is to be used in any data synthesis | 6-7 |
| Summary measures | 13 | State the principal summary measures (such as risk ratio, difference in means). | 3, 7 |
| Synthesis of results | 14 | Describe the methods of handling data and combining results of studies, if done, including measures of consistency (such as $I^2$ statistic) for each meta-analysis | 8 |
| Risk of bias across studies | 15 | Specify any assessment of risk of bias that may affect the cumulative evidence (such as publication bias, selective reporting within studies) | 8 |
| Additional analyses | 16 | Describe methods of additional analyses (such as sensitivity or subgroup analyses, meta-regression), if done, indicating which were pre-specified | 8-9 |
| **Results** | | | |
| Study selection | 17 | Give numbers of studies screened, assessed for eligibility, and included in the review, with reasons for exclusions at each stage, ideally with a flow diagram | 9 |
| Study characteristics | 18 | For each study, present characteristics for which data were extracted (such as study size, PICOS, follow-up period) and provide the citations | 10 |
| Risk of bias within studies | 19 | Present data on risk of bias of each study and, if available, any outcome-level assessment (see item 12). | S6 |
| Results of individual studies | 20 | For all outcomes considered (benefits or harms), present for each study (a) simple summary data for each intervention group and (b) effect estimates and confidence intervals, ideally with a forest plot | S5 |
| Synthesis of results | 21 | Present results of each meta-analysis done, including confidence intervals and measures of consistency | 11 |
| Risk of bias across studies | 22 | Present results of any assessment of risk of bias across studies (see item 15) | S5 |
| Additional analysis | 23 | Give results of additional analyses, if done (such as sensitivity or subgroup analyses, meta-regression) (see item 16) | 13-14, S3-4, S8 |

| Section/topic | Item No | Checklist item | Reported on page No |
|---|---|---|---|
| **Discussion** | | | |
| Summary of evidence | 24 | Summarise the main findings including the strength of evidence for each main outcome; consider their relevance to key groups (such as health care providers, users, and policy makers) | 14-15 |
| Limitations | 25 | Discuss limitations at study and outcome level (such as risk of bias), and at review level (such as incomplete retrieval of identified research, reporting bias) | 16 |
| Conclusions | 26 | Provide a general interpretation of the results in the context of other evidence, and implications for future research | 16-17 |
| **Funding** | | | |
| Funding | 27 | Describe sources of funding for the systematic review and other support (such as supply of data) and role of funders for the systematic review | 18 |

## Supplementary Information S2 - Search strategies and detailed records

| Relevant text of Population & Type | Relevant text of Intervention & Outcome |
|---|---|
| 1. B cell Leukemia<br>2. Lymphocytic leukemia<br>3. Chronic lymphocytic leukemia<br>4. Small lymphocytic leukemia<br>A= #1 or #2 or #3 or #4<br>5. Maintenance<br>6. Maintenance therapy<br>7. Consolidation<br>8. Consolidation therapy<br>B = #5 or #6 or #7 or #8<br>9. Trial<br>10. Clinical trial/ trials<br>11. Randomized<br>12. Randomization<br>13. Controlled trial/trials<br>14. Randomized controlled trial/trials<br>15. Controlled clinical trial<br>16. RCT<br>17. Persepctive study<br>18. Clinical study<br>19. Clinical article<br>C = #9 or #10 or #11 or #12 or #13 or #14 or #15 or #16 or #17 or #18 or #19<br>20. Progression free survival<br>21. Overall Survival<br>22. Response<br>23. Adverse Events<br>24. Mortality<br>D = #20 or #21 or #22 or #23 or #24 | 1. Lenalidomide<br>2. Revlimid<br>3. Linamide<br>E = #1 or #2 or #3<br>*. All search keyword with [Mesh Terms] or [All Fields]<br><br>**PUBMED**:<br>http://www.ncbi.nlm.nih.gov/pubmed<br><br>**EMBASE**:<br>https://www.embase.com<br><br>**COCHRANE CENTRAL**:<br>*https://www.cochrane.com* |

**Supplementary Information S3 - Assessment of risk of bias**

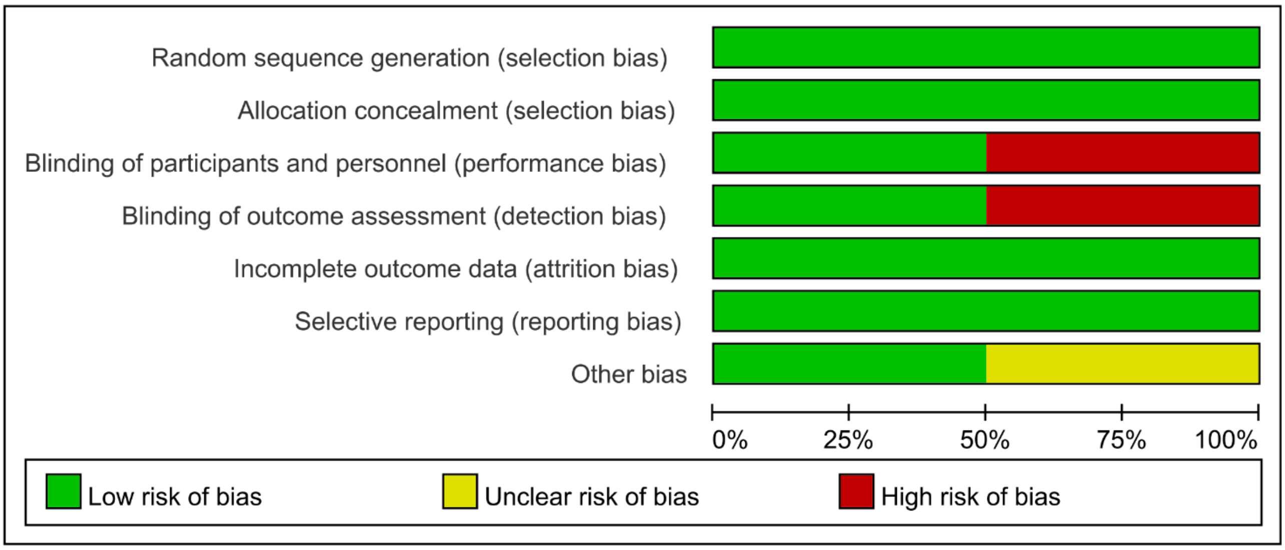


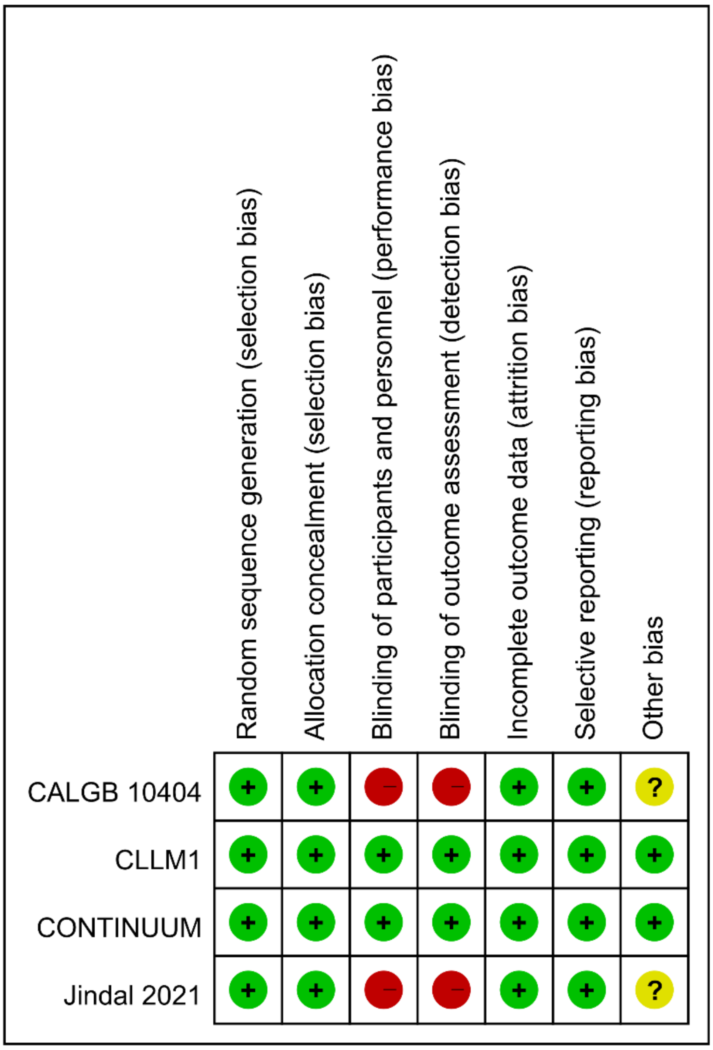

**Supplementary Information S4 - Secondary outcomes of meta-analysis.**

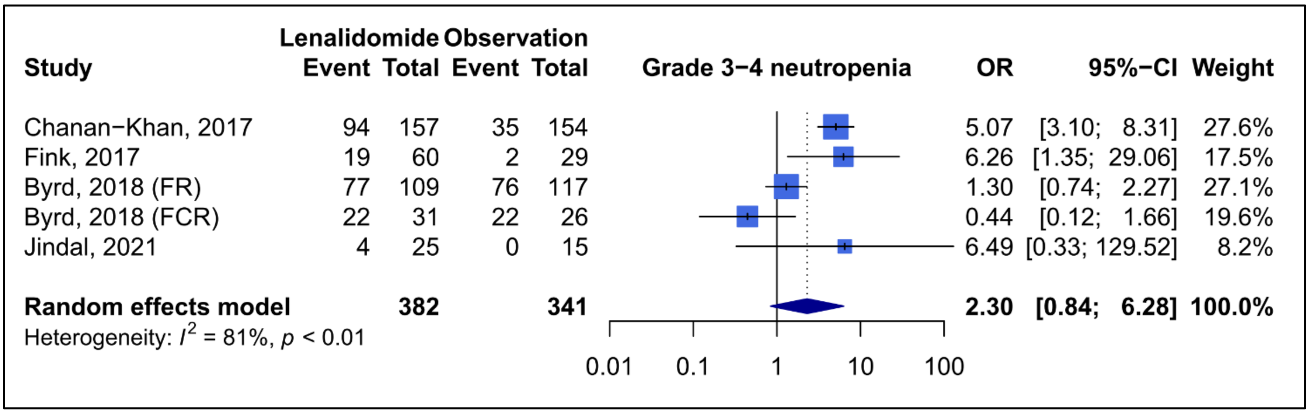


**Secondary outcomes of meta-analysis - Grade 3-4 neutropenia**

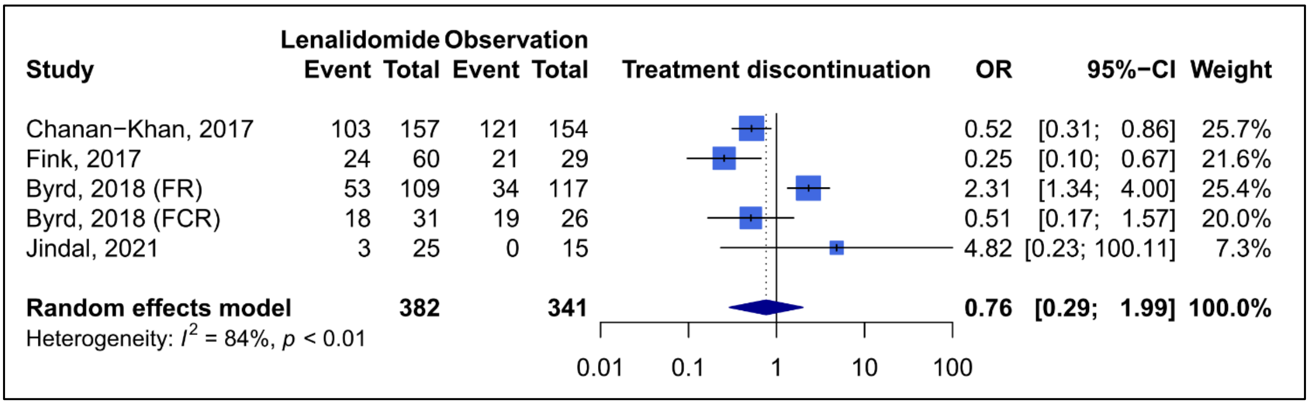


**Secondary outcomes of meta-analysis – Treatment discontinuation**

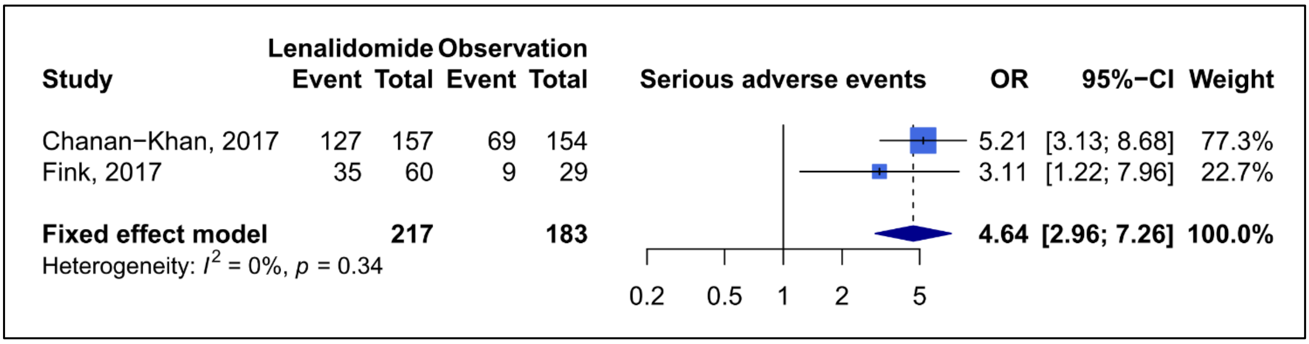


**Secondary outcomes of meta-analysis – Serious adverse events**

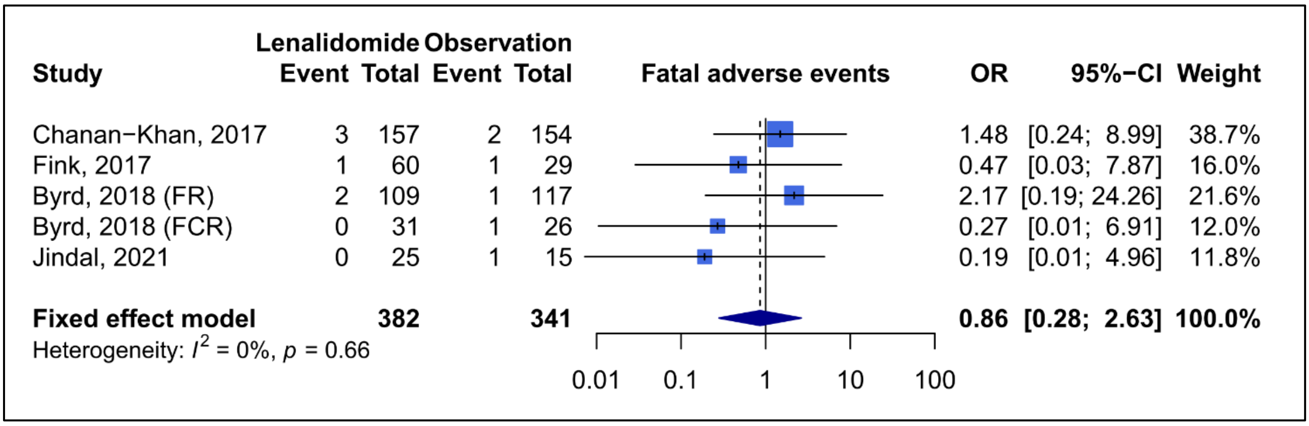


**Secondary outcomes of meta-analysis - Fatal adverse events**

**Supplementary Information S5 - Subgroup analysis of outcomes**

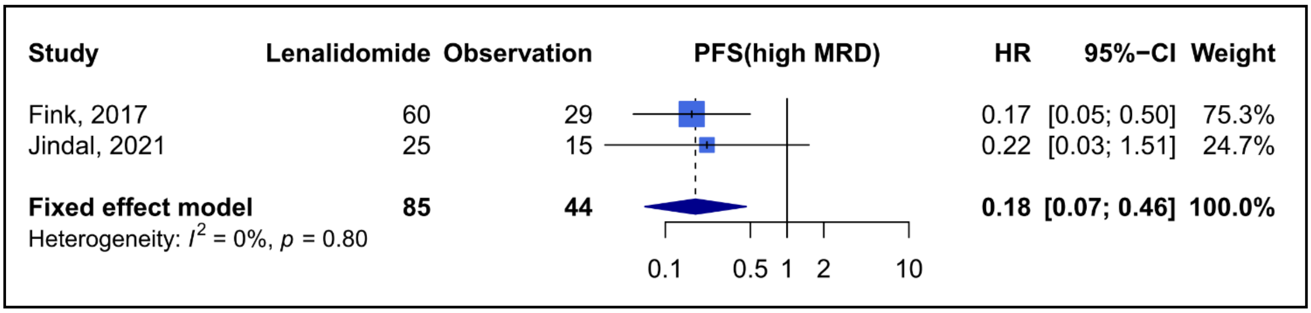


**Subgroup analysis of Progression-Free Survival (PFS) in patients with high minimal residual disease (MRD)**

The included patients were categorized with high minimal residual disease level. Outcome analyses were performed using hazard ratio with related 95% confidence intervals (95% CI).

**HR**, Hazard ratio; **CI**, Confidence intervals

**Supplementary Information S6 - Funnel plots and Egger's test**

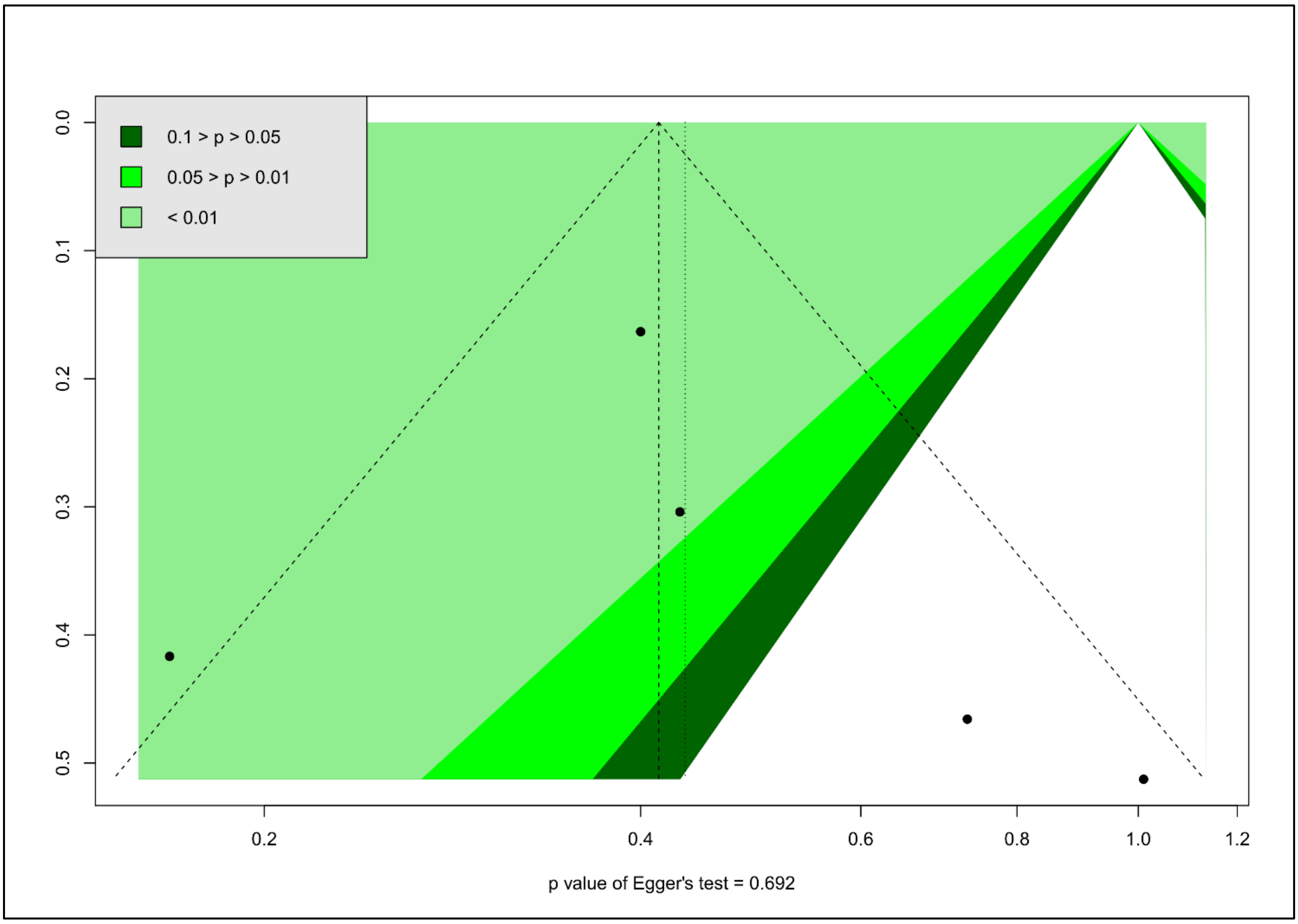


**Funnel plots and Egger's test in outcome for Progression-Free Survival (PFS)**

P-value: The significant level was set as 0.05;

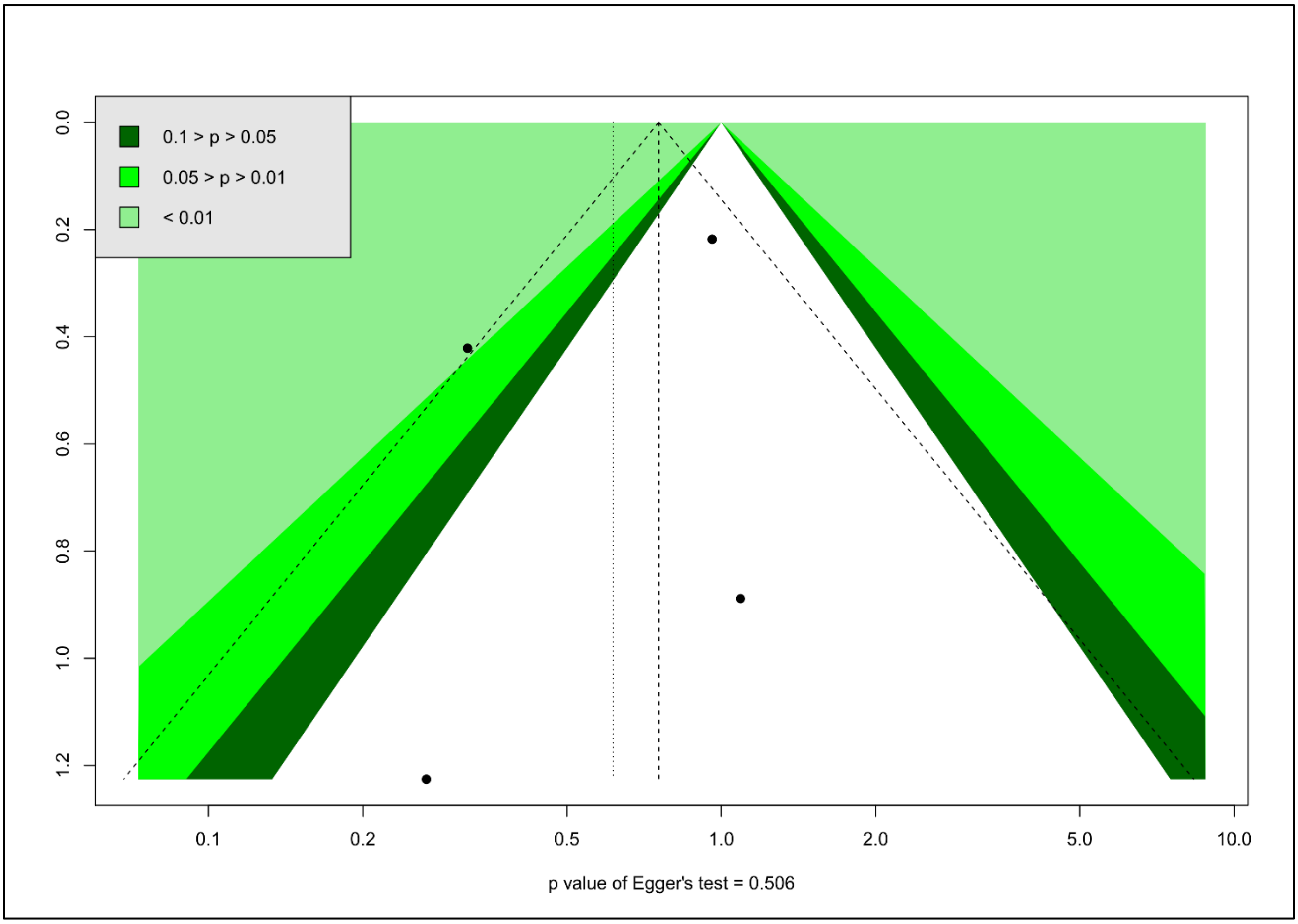


**Funnel plots and Egger's test in outcome for Overall Survival (OS)**

P-value: The significant level was set as 0.05;

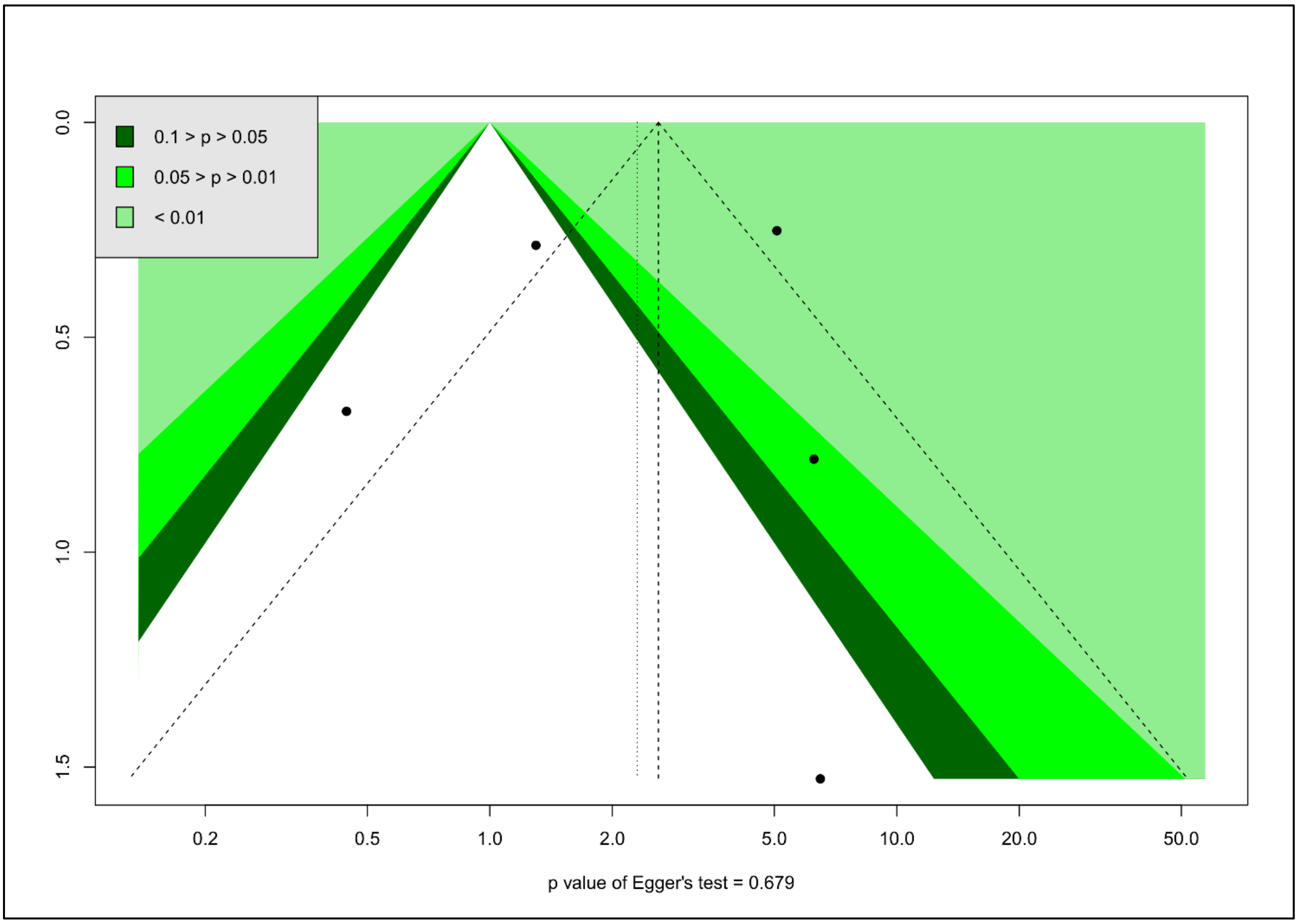


**Funnel plots and Egger's test in outcome for Grade 3-4 neutropenia**

P-value: The significant level was set as 0.05;

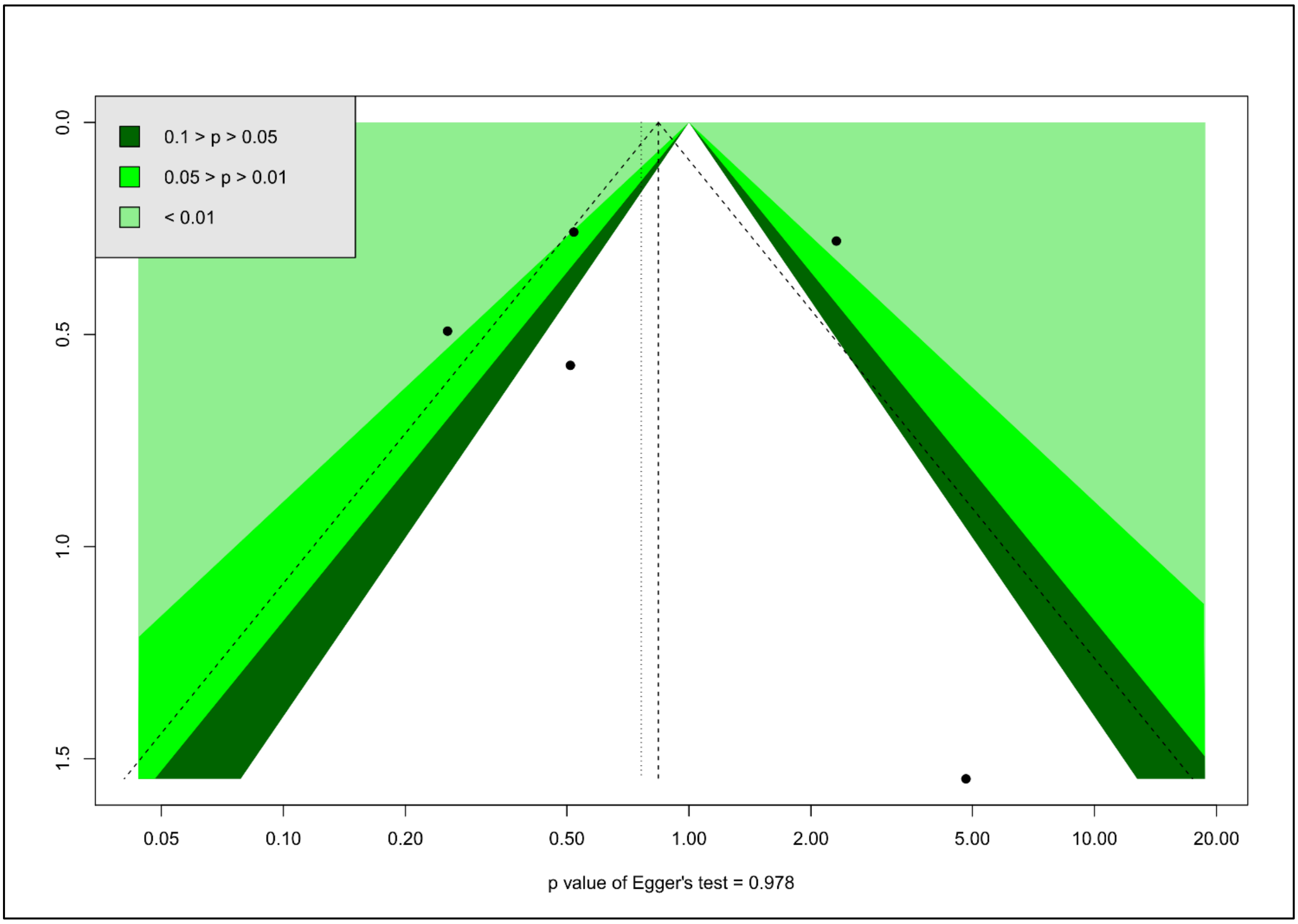


**Funnel plots and Egger’s test in outcome for Treatment discontinuation (TD)**

P-value: The significant level was set as 0.05;

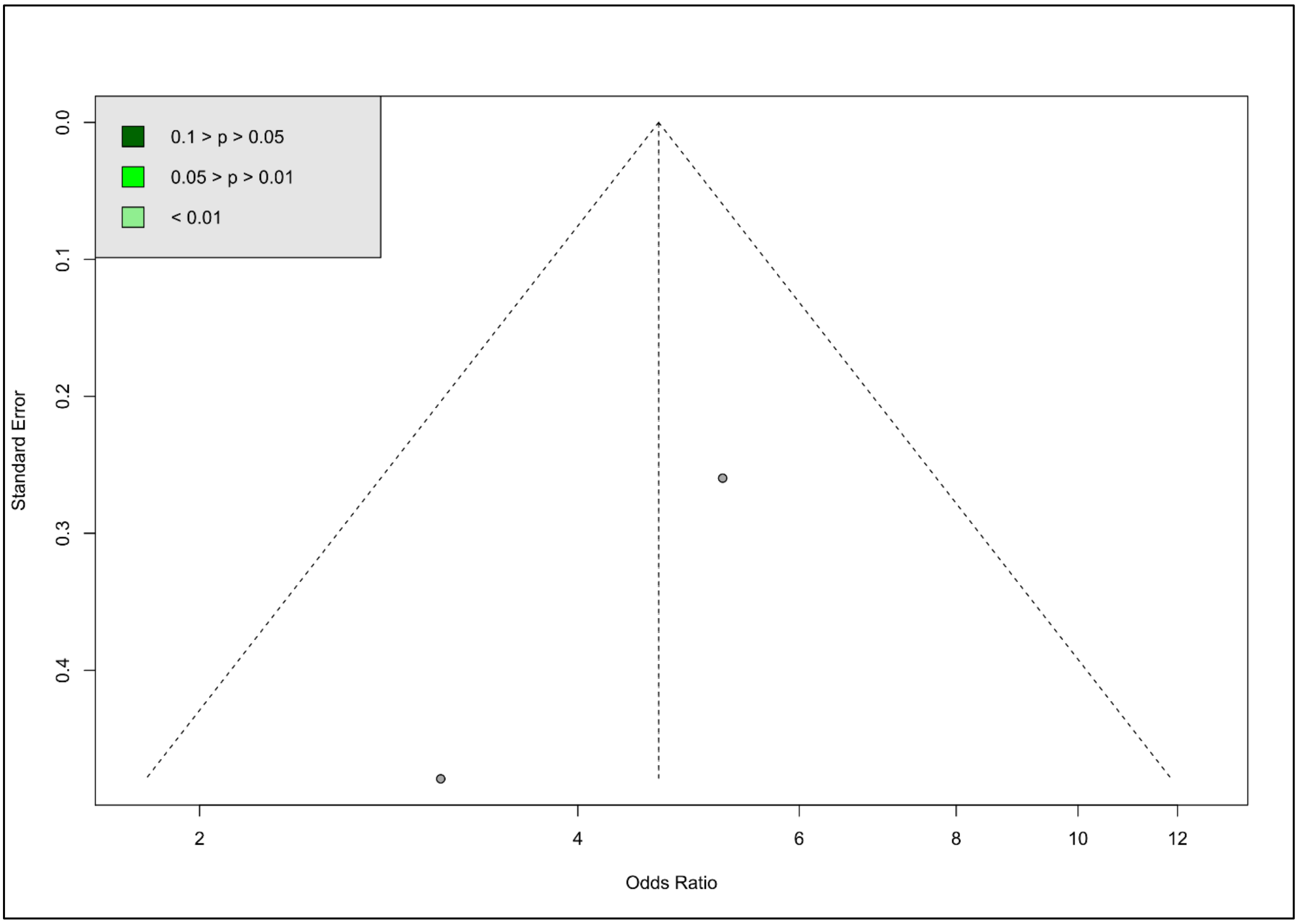


**Funnel plots and Egger’s test in outcome for Serious adverse events (SAE)**

P-value: The significant level was set as 0.05;

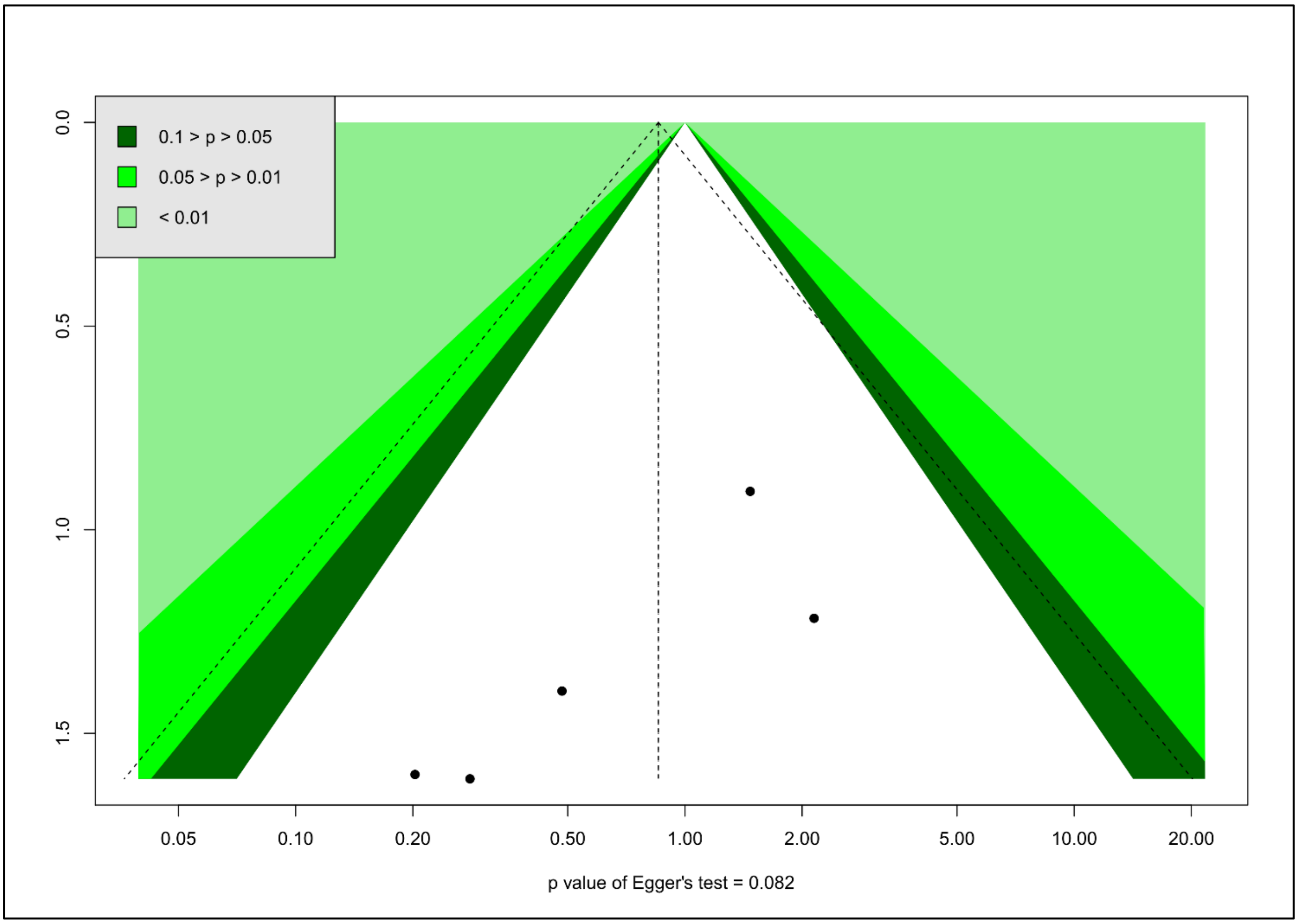


**Funnel plots and Egger's test in outcome for Fatal adverse events (FAE)**

P-value: The significant level was set as 0.05;

Note:

The funnel plots and Egger's test should be interpreted in caution, because the studies included in those outcomes were few in numbers.
